# Gas Sensor Based on 3-D WO_3_ Inverse Opal: Design and Applications

**DOI:** 10.3390/s17040710

**Published:** 2017-03-29

**Authors:** Ruiqing Xing, Yang Du, Xiaonan Zhao, Xiu Zhang

**Affiliations:** 1 Tianjin Key laboratory of Wireless Mobile Communications and Power Transmission, Tianjin Normal University, Tianjin 300387, China; tjuduyang@163.com (Y.D.); xiaonan5875@163.com (X.Z.); ecezhang@mail.tjnu.edu.cn (X.Z.); 2 College of Electronic and Communication Engineering, Tianjin Normal University, Tianjin 300387, China

**Keywords:** WO_3_, acetone, gas sensing, monitoring, inverse opal

## Abstract

A three-dimensional inverse opal (3DIO) WO_3_ architecture has been synthesized via a simple sacrificial template method. Morphology features of the 3DIO were characterized by scanning electron microscope (SEM) and its structure was characterized by X-ray diffraction (XRD). The shrinking ratio of the PMMA spheres was ~28.2% through measuring the distribution of the PMMA spheres and 3DIO WO_3_ center-to-center distance between the spheres and macropores, respectively. Beyond that, the 3DIO gas sensing properties were investigated systematically and the sensing mechanism of 3DIO WO_3_ was proposed. The results indicated that the response of the 3DIO sensor possessed excellent sensitivity to acetone gas, especially at trace levels. The 3DIO gas sensor response was ~7 to 5 ppm of acetone and could detect acetone low to 0.2 ppm effectively, which was in close proximity to the theoretical low detection limit of 0.14 ppm when R_a_/R_g_ ≥ 1.2 was used as the criterion for reliable gas sensing. All in all, the obvious satisfaction of the gas-sensing properties was ascribed to the structure of the 3DIO, and the sensor could be a promising novel device in the future.

## 1. Introduction

The Internet of Things has drawn considerable attention recently, and sensors, as the most important components of the Internet of Things, have been thoroughly researched [1]. Gas sensors have attracted significant attention with the environment attracting more attention [2]. Until now, many methods have been explored with respect to gas detection, [3,4,5,6,7,8,9,10] and metal oxide semiconductors have been extensively developed compared to others in the gas-sensing field, due to the advantages of high sensitivity, fast response and recovery, low detection limits, low fabrication cost, and simplicity in fabrication and measurement [11,12,13,14]. Additionally, it is generally known that the gas-sensing performance of oxide semiconductor sensors is strongly connected with the nanostructure morphology for the reactions of target gases and the sensing materials that are likely to take place at the sensing material’s surface. Therefore, the gas-sensing performance could be controlled effectively by changing the surface morphology of the oxide semiconductor materials [15,16].

Up to now, tremendous efforts have been devoted to designing different types of nanostructured morphologically-functional materials, such as electrospinning, solvothermal methods, sol-gel methods, magnetron sputtering, and vapor deposition methods. Although some progress has been made through controlling the pore size, surface area, porosity, and film thickness of the gas sensor, further exploration is necessary. The 3DIO semiconducting metal oxide nanostructure attracts more and more attention and research because of its near-ideal microstructures for gas sensors in recent years [17]. As a typical representative structure, the 3DIO structure usually shows a large surface area and a superior well-ordered porous architecture, all of which contribute significantly to plenty of active sites on the surface of the sensing materials and the effective reaction and diffusion of the target gas. As a consequence, these advantages make the 3DIO gas-sensing properties superior to other structures and many 3DIO semiconducting metal oxide nanomaterials have been prepared and studied [17,18,19,20,21]. Recently, Lee et al. prepared an n-type monolayer of 3DIO α-Fe_2_O_3_ and detected its sensing properties to NO_2_ [20]. The results showed that the prepared gas sensor demonstrates excellent gas sensing properties, besides showing a reversible switching from p- to n-type sensing property. Liang et al. successfully enhanced methanol gas-sensing properties though synthesis of 3DIO ABO_3_ perovskite-type oxides [22]. All of these studies indicated that the introducing of a 3DIO nanostructure might bring unique characteristics to the gas-sensing field.

Acetone, as a volatile organic compund (VOCs), exists extensively in our surrounding environment, such as building decoration materials, paint, glue, and in the laboratory, which is harmful to health and can lead to hazardous central nervous system dysfunction, bronchitis, and dermatitis. Beyond that, clinical data indicate that acetone concentrations in exhaled gas from healthy people are different from diabetics. Thus, real-time monitoring of acetone will be an extremely rewarding work in the environment and in health care. WO_3_, an important n-type semiconductor is extensively used in the gas sensor application area due to its advantages of a wide band gap, nontoxicity, and abundant sources [23]. Due to its physical and chemical properties, WO_3_ is widely applied to the analysis of H_2_, NO, NO_x_, NH_3_, acetone, H_2_S, etc. Thus far, a number of promising results have demonstrated the potential analysis of target gases using various nanostructures of WO_3_, such as nanorods, nanoflowers, nanowires, nanotubes, nanospheres, hollow spheres, and hierarchical structures. As one of the most used metal oxide gas sensors for the detection of acetone, WO_3_ sensors are capable of measuring acetone at the parts per billion (ppb) level, and could maintain a high signal-to-noise ratio in quite high relative humidity conditions, which is helpful to the detection of exhaled acetone gas [24]. Despite of all of the studies, to obtain high-performance WO_3_ gas sensors is still a challenge due to the controlling of WO_3_ nanostructures accurately with small crystals, and crafting superior surface properties is still a primary challenge for scientists. Considering the advantage of the 3DIO structure and the reported research of 3DIO WO_3_ is quite rare [25,26,27,28,29], in this paper, a sensor based on 3DIO WO_3_ is synthesized successfully and applied to acetone monitoring. The experimental results indicate that the 3DIO WO_3_ sensor demonstrates satisfactory gas-sensing performance and can monitor trace levels of acetone in a laboratory environment.

## 2. Experimental Section

### 2.1. Fabrication of WO_3_ Films

**Figure 1:**
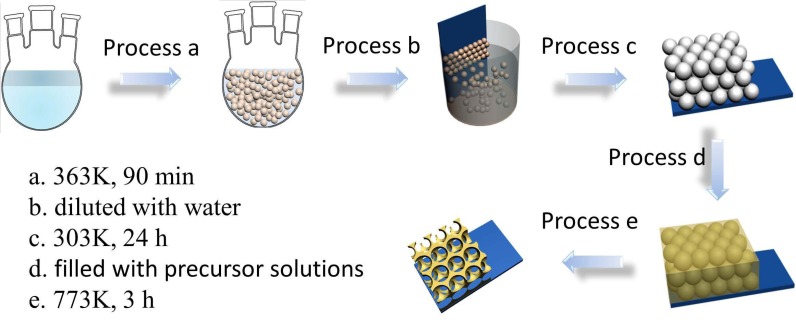
Schematic view of the process to fabricate the 3DIO WO_3_.

3DIO WO_3_ films were prepared by the sacrificial template method with a PMMA latex sphere opal as a template which was synthesized by methyl methacrylate [30,31]. The possible synthesis scheme of preparing the 3DIO WO_3_ films is shown in Figure 1 schematically, and descriptive processes were as follows: Firstly, the preparation of the PMMA spheres’ opal films: a stoichiometric amount of potassium persulfate was dissolved in deionized water before methyl methacrylate was added, and then the mixture was sintered for 90 min at 363 K in an oil bath to obtain PMMA latex spheres. Then, the obtained PMMA latex spheres were diluted with deionized water to form a 5% solid content colloid suspension. After that, the glass substrates, which were immersed into sulfuric acid for 2 h and washed with deionized water, were initially inserted into the aqueous colloid suspension vertically. Finally, the whole setup was placed in a 303 K oven for 24 h and then thin opal templates would self-assemble on the glass substrates with the evaporation of the water solvent. Secondly, the filling of the precursor solution: lacunas of the as-prepared thin opal templates were filled with the precursor solutions, which contained 0.08 M ammonium metatungstate hydrate and 0.26 M citric acid as a complexing agent by capillary force. Thirdly, the obtaining of the 3DIO WO_3_ films: the obtained films were placed in a tube furnace to remove the polymer spheres completely with a rising speed of 1 K/min to 773 K and sustained for 3 h, and the 3DIO WO_3_ films were finally obtained.

### 2.2. Characterization 

The morphology of the 3DIO WO_3_ film was inspected using a JEOL JSM-7500F field SEM (Tokyo, Japan) at an accelerating voltage of 15 kV with gold sputtered on the surface. The XRD patterns were conducted on a Rigaku D/max 2550 (Tokyo, Japan) using a monochromatized Cu target radiation resource (λ = 1.5045 Å) and the corresponding lattice constants of the samples were calculated by MDI Jade 5.0 software based on the XRD data. The gas-sensing properties were measured on a WS–30A system (Weisheng Instruments Co., Zhengzhou, China).

In this work, the different gas volume fractions of the target gases were obtained by the static distribution gas method, and the gas sources were 1000 ppm standard gas. The target gases’ preparation processes were as follows: Firstly, a 2.5 L volume glass chamber with stopper-rubber was pumped to a vacuum state. Then, a certain amount of the standard gas was injected into the glass chamber though a small mouth, followed by mixing with the atmospheric air. The injected amount of the standard gas can calculate by the following formula:

V_2_ = V_1_ × C_1_/C_2_ where V_2_ is the injected volume, V_1_ is the glass chamber volume, C_1_ (ppm) is the required gas concentration in glass chamber, C_2_ (ppm) is the 1000 ppm standard gas. For instance, 100 ppm (C_1_) required gas could be obtained by injecting 0.1 L (V_2_) 1000 ppm (C_2_) standard gas into the 1 L (V_1_) glass chamber.

When testing, the sensor with an extended line was put into the glass chamber, and then the sensor response reached a constant value. Thereafter, the sensor was removed from the glass chamber and placed in atmospheric air in order to recover to its original state. The response is defined as R_a_/R_g_ for an n-type sensor (R_a_ and R_g_ are the resistance of sensors in air and in the target gas, respectively). The response and recovery times are defined as the time required to reach 90% of the final equilibrium value.

### 2.3. Fabrication and Measurement of Gas Sensing Properties

**Figure 2:**
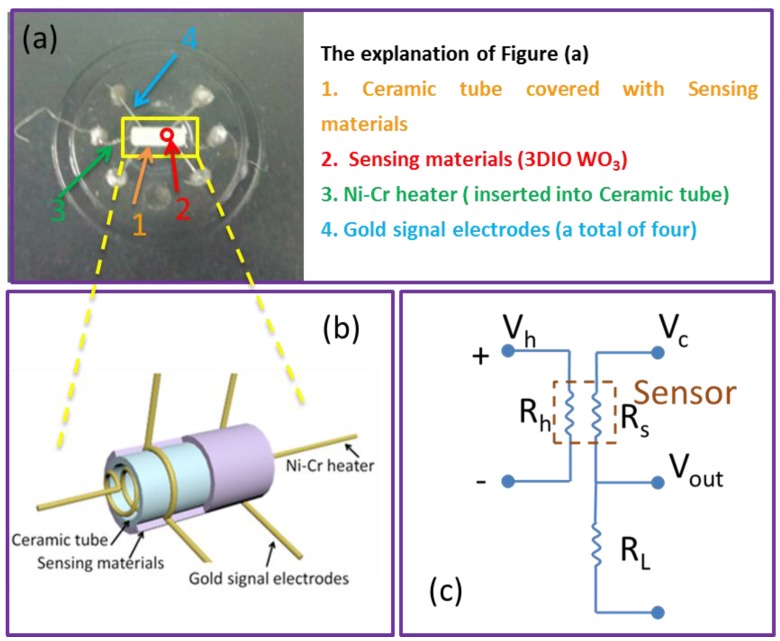
(**a**) Photograph of the 3DIO WO_3_ gas sensor, (**b**) the schematic structure of the gas sensor, and (**c**) the schematic diagram of the electrical circuit for measuring the 3DIO gas sensor.

The 3DIO WO_3_ films were scraped off from the substrates by a metal blade, and then the powder was mixed with ethanol in a weight ratio of 5:1 (*w*/*w*) to form a paste. Next, the paste was coated on an alumina tube (4 mm in length, 1.2 mm in external diameter, and 0.8 mm in internal diameter) on which a pair of gold electrodes was previously attached. After the solvent was evaporated, the ceramic tube with the thin layer was sintered in an oven at 623 K for 2 h in order to enhance its mechanical stability. Soon after, a spring-like nickel-chrome (Ni–Cr) alloy (28 Ω) heating wire was inserted into the alumina tube, ensuring both substrate heating and operating temperature. The heating wire and the electrodes of the alumina tube were then welded on a tailor-made support, as shown in Figure 2a. Subsequently, the gas sensor was thermally aged with a heating voltage of 5 V for five days before the first measurement. Figure 2a,b show a photograph and a schematic of the gas sensor, respectively. Figure 2c shows the schematic diagram of the electrical circuit for measuring the 3DIO gas sensor. V_h_ is the heating power, R_h_ is the resistance of the Ni–Cr alloy wire (during testing, the working temperature of the sensor was adjusted by controlling the heating voltage V_h_ of the Ni–Cr alloy wire, and this control process relies on the W-30 system), V_c_ is the 5 V circuit power, and R_L_ is the load resistance. The gas sensor properties in air or in the target gas are measured by monitoring the voltage output (V_out_) of the load resistance R_L_. All tests are performed under room temperature.

## 3. Results and Discussion

### 3.1. Morphological and Structural Characteristics

**Figure 3:**
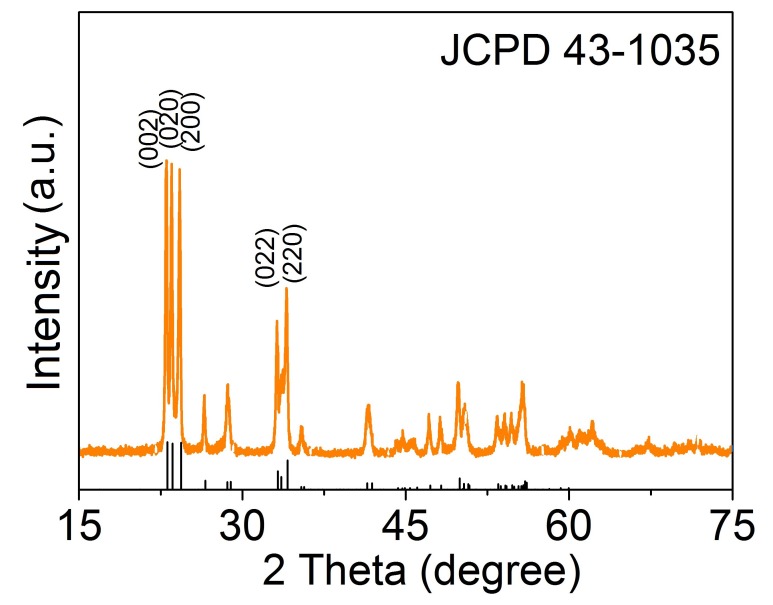
XRD pattern of the 3DIO WO_3_ sample.

XRD was first implemented to confirm the phase composition and crystallinity of the 3DIO films. As shown in Figure 3, XRD typical pattern of 3DIO films proved that the crystal phase of the films was WO_3_, and the diffraction peaks were readily indexed to (002), (020), and (200) reflections of the monoclinic WO_3_ phase, which agreed well with the reported values from the Joint Committee on Powder Diffraction Standards card (JCPDS card 43-1035). Moreover, no other diffraction peaks were observed, indicating the absence of other impurities.

**Figure 4:**
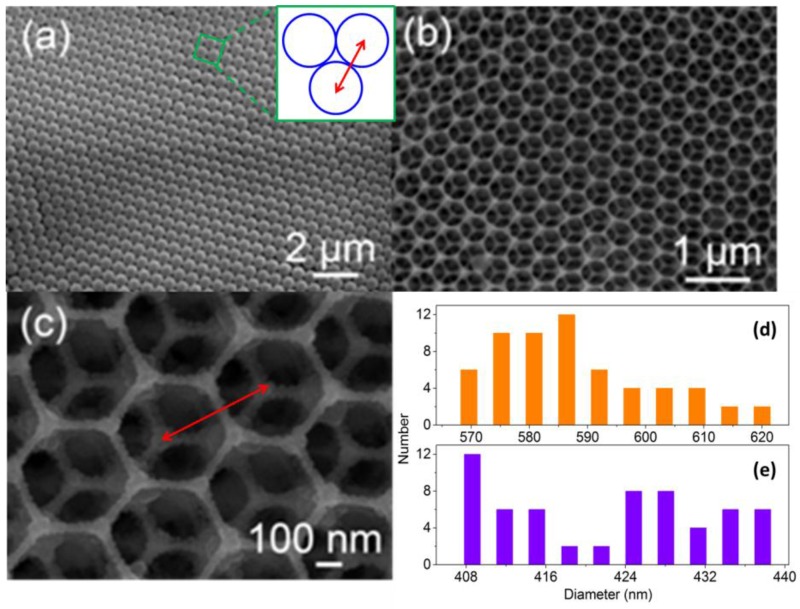
SEM images of (**a**) PMMA sphere opal films, (**b**) 3DIO WO_3_ sample, and (**c**) the enlarged SEM images of 3DIO WO_3_ on the glass substrate. (**d**,**e**) The size distribution of the PMMA spheres and 3DIO WO_3_ center-to-center distance, and the size distribution were estimated by measuring 60 cells. The inserted picture of (**a**) is the enlarged scheme of the PMMA in the green box. The red arrows in (**a**,**c**) revealed the center-to-center distances of PMMA spheres and 3DIO WO_3_, respectively.

The morphologies and microstructure of the as prepared PMMA spheres opal films and 3DIO WO_3_ films are characterized in Figure 4 by using SEM. Figure 4a,b reveal that both the as-prepared PMMA sphere opal films and 3DIO WO_3_ films yield well-defined ordered nanostructures in a large range. To further investigate the polycrystalline structural nature of the 3DIO in Figure 4b, the SEM image of the 3DIO is enlarged and displayed in Figure 4c, which provides similar information of Figure 4b. As can be clearly seen, the 3DIO structure is similar to the hexagonal structure of honeycomb, which is one of the most stable structures in nature. This architecture ensures no collapse from pore and pore, which contributes to the spreading of the target gas. Moreover, as the red arrow points out in Figure 4a,c, the distributions of the PMMA spheres and th e3DIO WO_3_ center-to-center distance between the spheres and macropores are measured and shown in Figure 4d,e, respectively. Figure 4d,e show that 3DIO center-to-center distance is much smaller than that of the PMMA spheres (center-to-center distances are ~588.1 nm and ~422.2 nm, respectively), which can be attributed to the shrinkage of sphere diameter during calcination. Based on Figure 4d,e, further computing indicates that the shrinkage of the sphere diameter is ~28.2%, which is within the shrinking ratio of PMMA spheres.

### 3.2. Gas Sensing Properties of 3DIO WO_3_ Sensors

**Figure 5:**
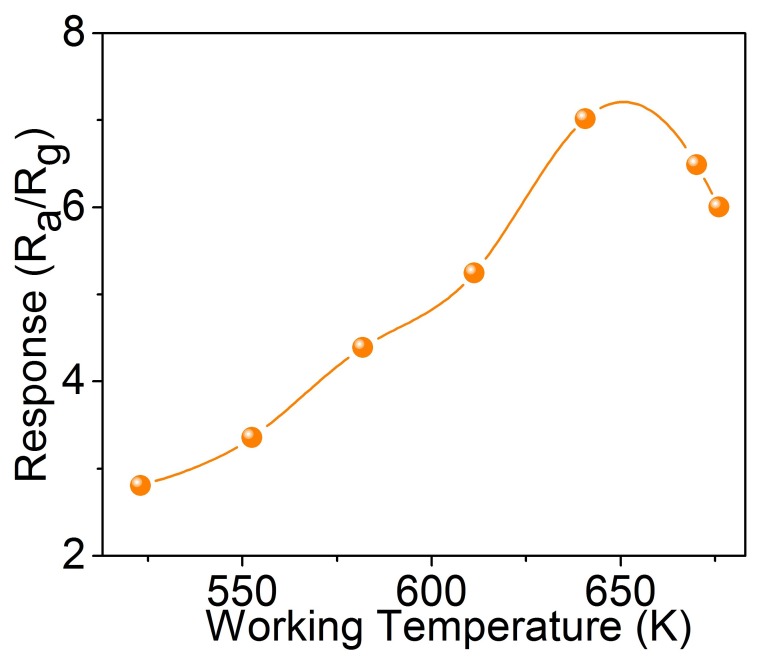
Relationship of responses vs. the operating temperature of the 3DIO WO_3_ gas sensors to 5 ppm acetone. The lines are spline curves.

The 3DIO nanostructure is a promising candidate as a gas sensor. The unique structure is capable of providing a large surface to volume ratio and good permeability, which is desirable for the adsorption and diffusion of target gases in sensor materials [17,22]. The 3DIO WO_3_ gas sensor was fabricated according to the above method and studied carefully. All of the sensors are placed under room temperature in an air atmosphere, and the whole testing process is also accomplished at room temperature. Considering the significant influence of the operating temperature on the sensitivity of gas sensors, the gas sensor was tested at different temperatures to determine the optimum operating conditions for acetone detection first [32]. Through controlling the heating voltage V_h_ of the Ni–Cr alloy wire in Figure 2c, various operating temperatures are acquired. Figure 5 depicts the response curve of the 3DIO WO_3_ gas sensor towards 5 ppm acetone gas as a function of the working temperature, and the lines are spline curves, which are used to show the relationships between the response and the working temperature vividly. It is observed that the 3DIO gas sensor shows typical n-type sensing characteristics—the response curve increases, at first, until reaching a maximum, and then decreases with the increase of the working temperature. Therefore, the gas sensor reacts most effectively at a particular temperature. In our case, the optimal working temperature is ~643 K and its corresponding response is ~7.0 for 5 ppm acetone.

**Figure 6:**
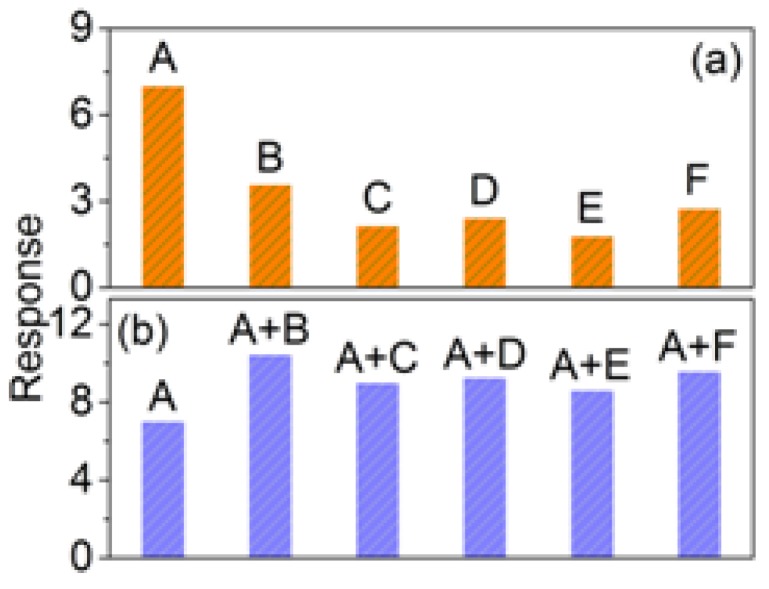
(**a**,**b**) Selective test of 3DIO WO_3_ sensor for 5 ppm target gases at ~643 K respectively (A = acetone, B = ethanol, C = methanol, D = methanal, E = toluene, and F = ammonia).

The selectivity indicator of the 3DIO gas sensor is tested by recording its responses at the working temperature of ~643 K to various testing gases, including ethanol, methanol, methanal, toluene, and ammonia at the concentration level of 5 ppm. The results shown in Figure 6a indicate that the 3DIO gas sensor shows the highest response to acetone, the lowest sensitivity toward methanol, and almost insensitivity to toluene. It is evident that the response of the 3DIO gas sensor to acetone against other gases exceeds two times, demonstrating the acceptable selectivity of the sensor to acetone. Moreover, the anti-interference performance of the sensor is tested in order to further investigate the gas sensor sensing performance indicators. As shown in Figure 6b, the responses are the sensor response when located in the mixed 5 ppm acetone and 5 ppm atmosphere of various gases. Comparing the response of Figure 6a,b, it is obvious that the response of the sensor when in acetone and another gas-mixed atmosphere is nearly equal to the response in acetone plus the response in another gas. In other words, the sensor response when in the mixed gases (gas one, gas two) is almost the same to the sum of the two responses when in gas one and gas two, respectively. This phenomenon could be attributed to complete reactions between the target gas molecules and adsorbed oxygen.

**Figure 7:**
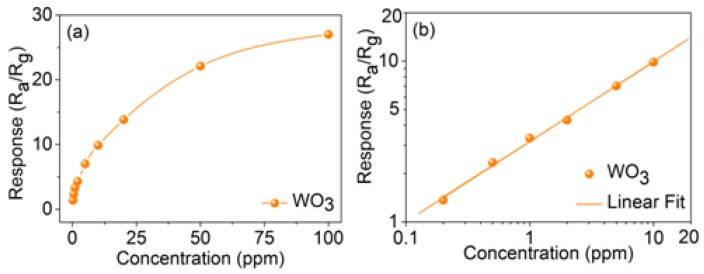
Responses of the 3DIO WO_3_ sensor to different acetone concentrations in the normal coordinate system (0.2–100 ppm) (**a**) and log-log coordinate system (0.2–10 ppm) (**b**).

Figure 7 shows the relationship of response vs. the acetone concentration ranging of 0.2–100 ppm at ~643 K. As can be seen, the response of the sensor increases dramatically when the acetone gas concentration is lower, and increases slowly, even saturated, when the acetone concentration is higher, which can be ascribed to the incomplete reactions between acetone and the 3DIO sensitive material of the sensor. With the acetone concentration increasing, a mass of acetone is covered on the surface of the semiconductor oxide sensing materials leading to a lower surface reaction which then leads to the slowly increasing speed of the response. Clinical data indicate that the exhaled acetone of diabetes exceeded 1.8 ppm, while for healthy individuals is only 0.3–0.9 ppm [33,34]. Moreover, detection of low concentrations of acetone is of great significance for environmental safety. Thus, more attention is focused on the gas sensing performance when detecting low concentrations of acetone, and the corresponding response of the low gas concentration in Figure 7a is enlarged, as shown in Figure 7b for convenience. As can be clearly seen, the response of the 3DIO gas sensor is positively proportional to the growth of the acetone concentration in the log-log plot. Note that the 3DIO gas sensor clearly shows response signals even when acetone concentration is as low as 0.2 ppm, which is in close proximity to the deduced low detection limit of acetone (0.14 ppm) when Ra/Rg ≥ 1.2 is used as the criterion for reliable gas sensing. Note that the experimental testing detection limit and the theory detection limit are all lower than the clinical data concentration of exhaled acetone, demonstrating the satisfactory performance of the 3DIO gas sensor. In addition, the slope in Figure 7b is calculated to be ~0.49 (nearly 0.5) stating the existence of macropores in the 3DIO sensing material, and the detailed explanations are shown in the literature published previously [28]. Furthermore, considering the scientific and reasonable evaluation of the performance of the sensor, the response, actual detection limit, and response (τ_res_) and recovery (τ_rec_) times, the sensing properties of the as-prepared 3DIO WO_3_ gas sensor in this study are compared with some other reported metal oxide semiconductor gas sensors and the results are demonstrated in Table 1 [35,36,37,38,39,40]. As the table shows, the overall performance of the 3DIO WO_3_ gas sensor is at a satisfactory level, indicating that the 3DIO architecture is beneficial in improving gas sensing properties and might be applied in the gas sensing field.

**Figure 8:**
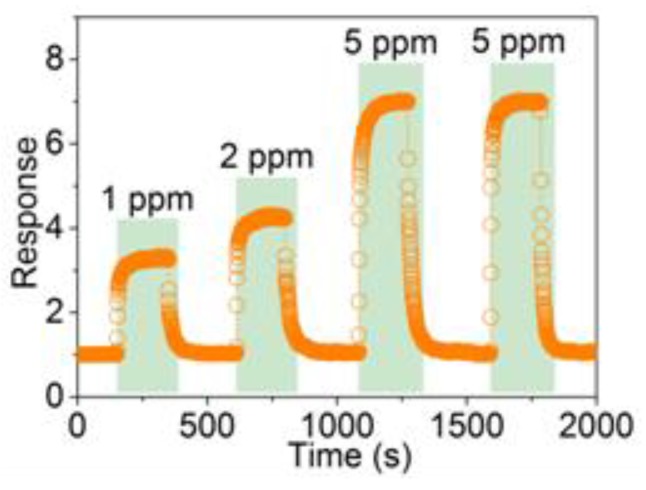
Four period response curves of the 3DIO WO_3_ sensor to different acetone concentrations.

In addition, the response performance of the 3DIO is further investigated at the working temperature of ~643 K. Figure 8 shows four dynamic processes of the sensor when exposed to different and the same acetone concentrations. As shown, the sensor responses all increased quickly when exposed to acetone gas and returned to its initial state when withdrawn from acetone gas within the four cycles. Additionally, the 3DIO sensor response increases linearly with the increase of acetone concentration, and is almost the same when detecting an identical concentration acetone gas. Based on the dynamic response curves of 5 ppm in Figure 8, the corresponding τ_res_ and τ_rec_ of the 3DIO sensor are calculated to be ~10 and 34 s, respectively.

### 3.3. Sensing Principle of the 3DIO WO_3_

For the WO_3_-based gas sensing device, its acetone sensitivity performance is essentially similar to that of H_2_ [3,31,41]. In our case, when acetone is let in, H atoms are firstly dissociated from acetone molecules on the surface of the 3DIO, which then transform into H^+^ ions with the liberation of an electron e^−^, resulting in H^+^. Then, when two H^+^ approach an oxygen ion of WO_3_, the band between the W ion and the O ion will be weakened and then a localized H_2_O molecule can form. Upon formation of the localized H_2_O, two e^−^ are released, which could lead to the decrease of the depletion region width and, consequently, the resistance of the film decreases. The progress can be described as follows:

WO_3_ + 2H^+^ + 2*x*e^−^ ⇌ WO_3−*x*_·*x*H_2_O (1)

Due to the gas sensor working temperature being quite high, it is possible that WO_3__−*x*_·*x*H_2_O breaks down into WO_3__−*x*_ (WO_3_ with oxygen vacancies) and H_2_O vapors, as follows:

WO_3−*x*_·*x*H_2_O ⇌ *x*H_2_O + WO_3−*x*_ (2)

When the sensor is exposed to the air environment, oxygen vacancies will be occupied and WO_3_ will form, as follows:

2WO_3−*x*_ + *x*O_2_ ⇌ 2WO_3_ (3)

Moreover, the satisfactory performances of the 3DIO gas sensor can be understood from two primary factors. Firstly, the slope in the log-log plot in Figure 7b is very close to 0.5, indicating the well-ordered porous architecture and almost no collapse and blocking of the 3DIO structure, which significantly contributes to the target gas diffusion and the charge transportation. In addition, the 3DIO possesses high surface-to-volume ratio and, thus, leads to complete reactions between the target gas molecules and the sensitive material. Secondly, Knudsen diffusion and molecular diffusion are possible owing to the unique 3DIO architecture showing both mesopores and macropores [42]. Therefore, the target gas could diffuse to the inner and the surface regions of the sensing films, which allows full reactions between the target gas and the sensing material, leading to the improvement of gas-sensing performance, accordingly.

## 4. Conclusions

In summary, 3DIO WO_3_ with monoclinic phase is prepared through a simple sacrificial template method and its gas-sensing properties are systematically investigated. In addition, the gas-sensing mechanism of the 3DIO architecture is discussed. The sensing results indicate that 3DIO WO_3_ shows satisfactory gas sensing performances to acetone gas, which could be attributed to the specific 3DIO structure with a large surface-to-volume ratio and excellent permeability. The response of the 3DIO sensor could up to ~7 when detecting 5 ppm acetone and the actual detection limit is 0.2 ppm, which is in close proximity to the theoretical low detection limit (0.14 ppm). Of particular note is both the experimental testing detection limit and the theoretical detection limit are all lower than the clinical data concentration of exhaled acetone, demonstrating that the as-prepared 3DIO sensor shows satisfactory gas-sensing properties and might be a promising acetone gas sensor used in environmental monitoring and noninvasive detection of illnesses in the future.

## Figures and Tables

**Table 1:** Comparison of the acetone gas sensing performances of some reported metal oxide sensors and the 3DIO WO_3_ sensor in this study.

Sensing Materials	Response (R_a_/R_g_)/Corresponding Concentration	Actual	Temperature	Response/Recovery Time	Reference
3DIO WO_3_	3.3/1 ppm	0.2 ppm	643 K	~10/34 s	This work
Co_3_O_4_ pure	1.3/10ppm	-	433 K	~48/18 s	[33]
SnO_2_@Co_3_O_4_	2.7/10 ppm	-	413 K	~35/78 s	[34]
WO_3_	56/150 ppm	-	473 K	~32/45 s	[35]
WO_3_ nanorods	3.1/0.5 ppm	0.25 ppm	503 K	~9/14 s	[36]
ZnO nanorod arrays	30.4/100 ppm	1 ppm	573 K	~5/15 s	[37]
Graphene- ZnFe_2_O_4_	3.5/10 ppm	1 ppm	548 K	~4/18 s	[38]
